# Femoral shortening does not impair functional outcome after internal fixation of femoral neck fractures in non-geriatric patients

**DOI:** 10.1007/s00402-018-3011-0

**Published:** 2018-07-27

**Authors:** Thomas Haider, Jakob Schnabel, Julian Hochpöchler, Gerald E. Wozasek

**Affiliations:** Department of Orthopedics and Trauma Surgery, Medical University of Vienna, Waehringer Guertel 18-20, 1090 Vienna, Austria

**Keywords:** Hip fracture, Femoral neck fracture, Femoral shortening, Internal fixation, Non-geriatric patients, Leg length discrepancy

## Abstract

**Introduction:**

Aim of this study was to investigate the incidence and extent of femoral shortening in non-geriatric patients after internal fixation of femoral neck fractures in relation to the clinical outcome at mid-term follow-up.

**Materials and methods:**

Reviewing our admission data, we identified non-geriatric patients (18–65 years) with femoral neck fractures treated with either dynamic hip screw or cancellous screws between 2007 and 2015. Patients were then contacted and invited to a follow-up clinical investigation including whole-leg standing X-rays.

**Results:**

A total of 40 patients with a mean age at surgery of 52 ± 9 years returned for the follow-up examination. Overall, 31 patients (77.5%) had undergone a dynamic hip screw fixation, while 9 patients were treated with cancellous screws (22.5%). The median follow-up time was 65.5 months (5.5 years). We observed shortening of the ipsilateral femur neck in the majority of cases (92.5%). Still, functional outcome in the overall study population was excellent with a median Harris Hip Score of 96.

**Conclusions:**

Femoral neck shortening is common in non-geriatric patients after internal fixation of femoral neck fractures. Nonetheless, observed excellent functional outcome at mid-term follow-up supports joint-preserving strategies in non-geriatric femoral neck fractures.

## Background

Femoral neck fractures are most common in daily orthopaedic trauma practice. Worldwide, 1.6 million patients suffer from femoral neck fractures every year [1, 2]. Due to higher prevalence of osteoporosis and tendency to fall, this type of fracture affects mainly geriatric patients [1]. Corresponding to global demographic development, a further increase of femoral neck fractures is expected [3]. Between 3 and 10% of hip fractures occur in young patients after more severe forms of trauma such as motor vehicle accidents or falls from great height [4]. Due to higher functional demands of young patients compared to geriatric patients, joint preservation with osteosynthesis is the treatment of choice. As standard treatment both cannulated screws as well as the dynamic hip screw (DHS) are well-established with satisfying results. Especially in cases of displaced femoral neck fractures with increased risk of avascular femoral head necrosis and high re-operation rates, primary hip replacement as initial treatment strategy delivers satisfying results in geriatric patients [5]. However in young patients, primary arthroplasty as first line treatment of femoral neck fractures remains controversially discussed [6, 7].

Fracture healing in general is associated with shortening of the affected bone. Factors such as compression, impaction and resorption at the fracture site result in shortening. Shortening of bones in the lower extremity leads to leg length discrepancy (LLD) causing pain, osteoarthritis, gait disturbance and impaired mobility [2, 8]. Due to lower functional demands and reduced mobility prior to the fracture, LLD following femoral neck fracture fixation in geriatric patients is often negligible [6, 9]. On the other hand, posttraumatic LLD can lead to major long-term complaints in young patients with long life expectancy [2, 8].

To our knowledge, there are no mid-to-long-term follow-up data available regarding femoral shortening in non-geriatric patients with femoral neck fractures after internal fixation. Also, data regarding functional outcome in relation to femoral neck shortening are lacking in the current literature. The aim of the study was to evaluate the incidence and extent of femoral shortening in relation to the clinical outcome in non-geriatric patients with femoral neck fractures treated with osteosynthesis.

## Methods

The study protocol was approved by the local ethics committee. We reviewed the admission data of our Level I trauma department and identified non-geriatric patients (18–65 years) with femoral neck fractures treated with either DHS or cancellous screws between 2007 and 2015 (*n* = 163). Exclusion criteria included no available contact information, pathological fracture, incomplete postoperative clinical records and/or radiographs, concomitant or previous surgery of the ipsilateral and/or contralateral femur, and secondary arthroplasty. A total of 48 patients met the inclusion criteria and were contacted. Forty patients returned for a follow-up investigation and gave their written informed consent to participate in the present study, while 8 patients declined to participate.

We then established a database including patient demographics, injury characteristics (type, mechanism, etc.), fracture type according to the AO/OTA classification, Garden classification and Pauwels classification, type of implant, time of surgery, interval between surgery and trauma, body mass index (BMI), as well as data from clinical and radiographic follow-up.

During the invited follow-up investigation, whole-leg standing X-rays were obtained. Clinical examination included recording of the range of motion (ROM) of both hips, the Harris Hip Score (HHS), the Staffelstein Score, the visual analogue scale for pain (VAS) in the affected hip, and the UCLA activity score. Values above 90 in the HHS were considered excellent, between 80 and 90 were defined as good results, while scores between 70 and 80 were graded as fair results, and measures of below 70 were defined as poor outcome.

Available postoperative X-rays were assessed for shortening of the femoral neck. We measured the length of the femoral neck in the axis of the center–collum–diaphysis (CCD) angle, as well as the distance between the center of the femoral head and the caudal end of the lesser trochanter (coined as “femoral shortening”) on both sides. Whole-leg standing X-ray was assessed using the same measurements, including measurement of the height difference between both acetabulum roofs (termed “leg length discrepancy”, “LLD”). Other possible causes of LLD (e.g. previous fractures) were further excluded in these radiographic studies.

### Surgical procedure and postoperative protocol

**Fig. 1:**
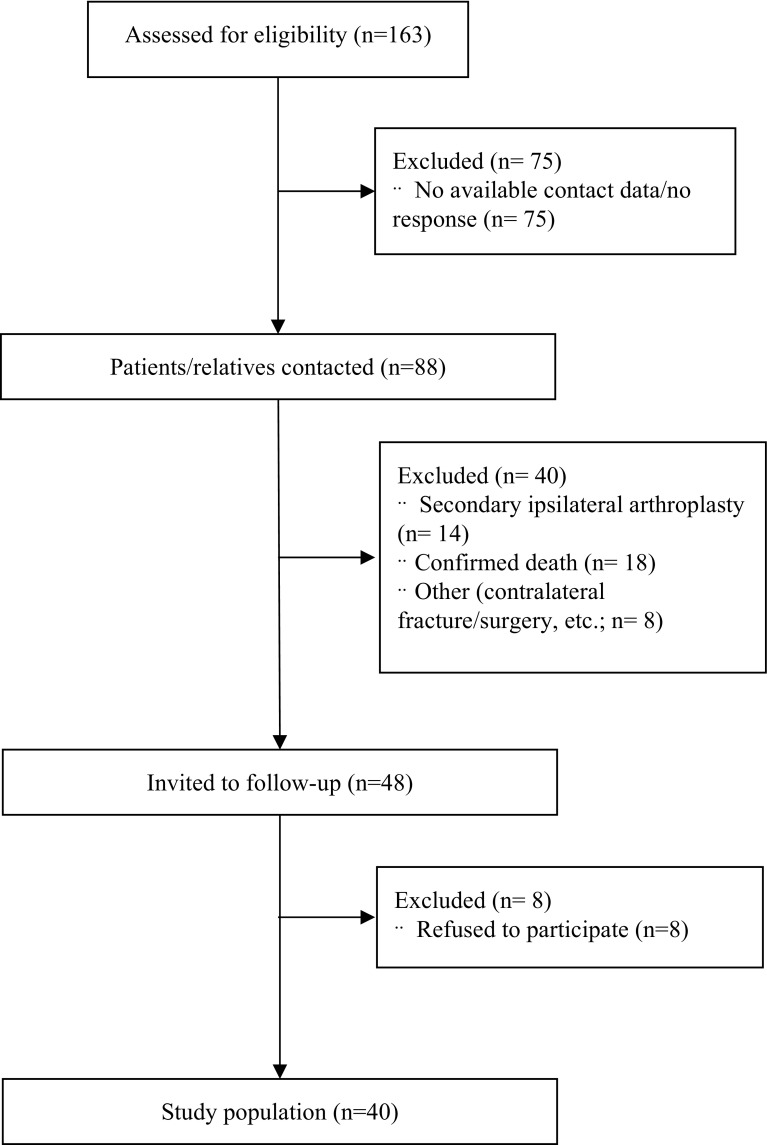
CONSORT diagram of recruited patients

Patients were positioned supine on a fracture table following general or spinal anaesthesia. All patients received single-shot antibiotic prophylaxis prior surgery. In all cases, closed reduction on the fracture table under fluoroscopic control in two planes was performed. We used cannulated screws from SanatMetal® and DHS implants from Depuy-Synthes®. Implantations were performed according to the manufacturer’s brochure under intraoperative fluoroscopic control. Pain medication was provided at the ward according to patient’s needs. Thrombosis prophylaxis with weight-adjusted low-molecular weight heparin was started 12 h after surgery and administered daily until full-weight bearing was allowed and feasible in all cases. The postoperative mobilisation scheme with non-weight bearing for 3 months after surgery was the same for both treatment groups. For this study, radiographic studies 3 months after surgery and X-rays performed at the invited follow-up investigation were examined (Fig. 1).

### Statistics

For all statistical calculations, we used SPSS Statistics (Version 23, IBM, New York, USA), while figures and graphs were drawn with GraphPad Prism 5 (GraphPad Software, San Diego, USA).

We compared the median of metric variables between the groups of interest with the Mann–Whitney–Wilcoxon rank-sum test or the Kruskal–Wallis test where appropriate. The student’s *t* test was used to compare mean values of variables with normal distribution. To compare categorical variables the Fisher’s exact test was utilized.

Analysis of potential correlation was conducted using Spearman’s non-parametric correlation.

**Fig. 2:**
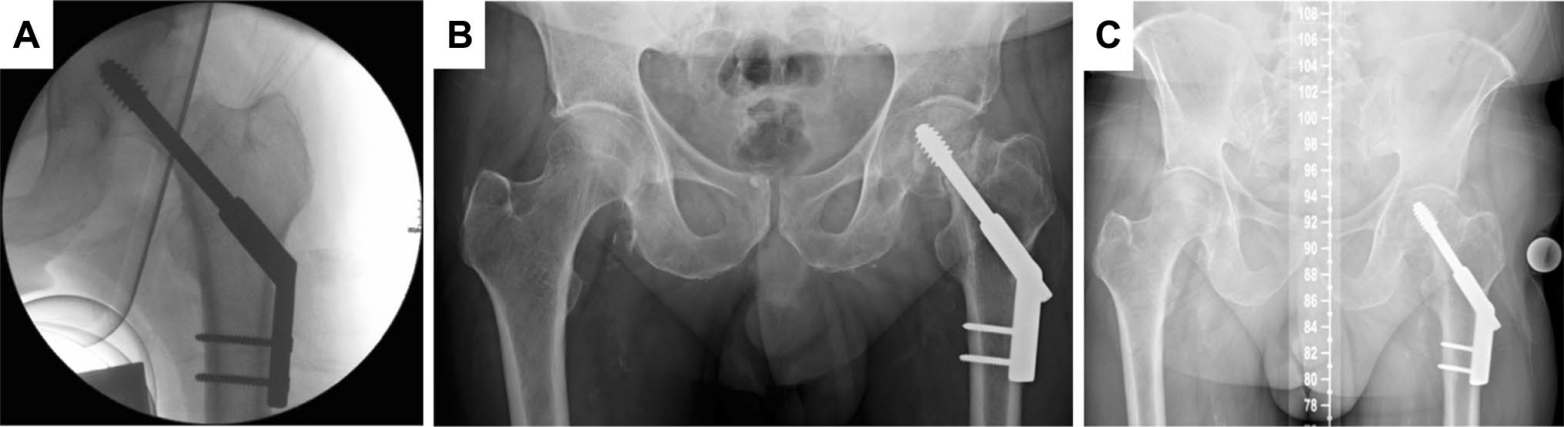
Patient (58 years) with medial femoral neck fracture and treatment with dynamic hip screw (DHS). **a** Intraoperative fluoroscopic image. **b** Follow-up x-ray 1 year after surgery. **c** Invited follow-up 6 years after surgery with leg length discrepancy (LLD) of 16 mm

We considered a *p* value < 0.05 to be statistically significant. If not stated otherwise, values are presented as median or, if appropriate, as mean ± standard deviation where appropriate. Interquartile range (IQR) was calculated and stated where appropriate (Fig. 2).

## Results

### Patient characteristics

Eighty-eight patients (54%) or their relatives were contactable. A total of 14 patients (16%) had received a secondary arthroplasty, while 18 patients (21%) had died.

Of the included 40 patients, 31 (77.5%) were treated with a DHS and 9 patients (22.5%) with cannulated screws. There were 19 female (47.5%) and 21 male patients (52.5%). The mean age at the day of surgery was 52 ± 9 years and 59 ± 9 years at the day of follow-up. The median follow-up time in months was 65.5 (5.5 years; IQR: 49.5–91.0). The most common cause of injury was fall (*n* = 22, 53.7%), followed by sport injuries (13, 31.7%) and traffic accidents (5, 12.2%). The fracture type was 31-B1 in 15 cases (41.7%), 31-B2 in 15 patients (41.7%), and 31-B3 in 6 patients (16.7%). According to the Garden classification, 12 patients (30%) suffered from a Garden I fracture, 14 patients (35%) sustained a Garden II fracture, while in 10 patients (25%) a Garden III fracture and in 4 patients (10%) a Garden IV fracture was seen. Displaced fractures (Garden III and IV fractures) were more often treated with DHS (*n* = 13 vs. 1). The median interval between injury and surgery was 5.5 h (min.-max.: 2.0–312.0 h). Average surgery time was significantly longer in the DHS group with 64 ± 25 min compared to 39 ± 13 min in patients treated with cannulated screws (*p* < 0.01; overall: 58 ± 25 min). Surgical time correlated directly with fracture type according to the Garden classification (*r* = 0.474, *p* < 0.01), but not with AO/OTA fracture type (*r* = 0.258, *p* = 0.064). The median hospitalisation time was 11 days (min.-max.: 5–86 days), comparable in both treatment groups (10 vs. 13 days, *p* = 0.517) and among patients with the three different fracture types according to AO/OTA (12 vs. 14 vs. 9 days, *p* = 0.077). Interestingly, time of hospitalisation was significantly longer in females compared to male patients (12 vs. 10 days, *p* < 0.05), although male patients sustained more dislocated fractures (Table 1).

**Table 1:** Demographic data of the study population

Characteristics	All patients (*n* = 40)	DHS (*n* = 31)	Cannulated screws (*n* = 9)	*P* value
Mean age (y)	53 (± 9)	52 (± 9)	58 (± 6)	**0.043**
Male (%)	21 (52.5)	19 (61.3)	2 (22.2)	0.06
Follow-up (m)	67.85	66.03	74.11	0.524
Time to surgery (h)	5.5	5.25	7.5	**0.02**
Surgery time (min)	55	60	35	**0.002**
Hospitalisation (d)	13	10	13	0.526
Fracture classification	–	–	–	–
Garden I	12	7	5	–
Garden II	14	11	3	–
Garden III	10	9	1	–
Garden IV	4	4	0	–

*y* years, *m* months, *h* hours, *min* minutes, *d* days, *LLD* leg length discrepancy, *mm* millimetres, *HHS* Harris Hip Score

Bold = *p* value < 0.05

On appointed follow-up, we observed avascular femoral head necrosis in one female patient (2.5%) who had sustained a B1 fracture which was initially treated with a DHS.

### Femoral shortening and leg length discrepancy

#### Short-term radiographic evaluation

Overall, in 37 patients (92.5%) femoral neck shortening and in 32 patients (80%) femoral shortening was observed 3 months after surgery. At 3 months postoperatively, the median femoral neck shortening was 7 mm (IQR: 3–11 mm, min–max.: 0–25 mm), while femoral shortening of the affected side was 5 mm (IQR: 2–8 mm, min–max.: 0–16 mm). Extent of shortening did not differ between gender, fracture type, trauma mechanism or treatment groups. Interestingly, fractures with higher Pauwels grade lead to a significantly larger extent of femoral shortening (3 vs. 4 vs. 7 mm, *p* < 0.05). Time of surgery and hospitalisation time did not correlate with neither extent of femoral neck shortening nor with femoral shortening.

#### Radiological follow-up at 5 years

At invited follow-up (median: 65.5 months), extent of shortening in general did not further increase. The median shortening of the femoral neck was 5 mm, while the observed median femoral shortening was 6 mm. In nine patients (22.5%) femoral neck shortening of more than 10 mm was seen, while femoral shortening of more than 10 mm was measured in ten patients (25%). All measurements did not differ between gender and treatment groups.

**Fig. 3:**
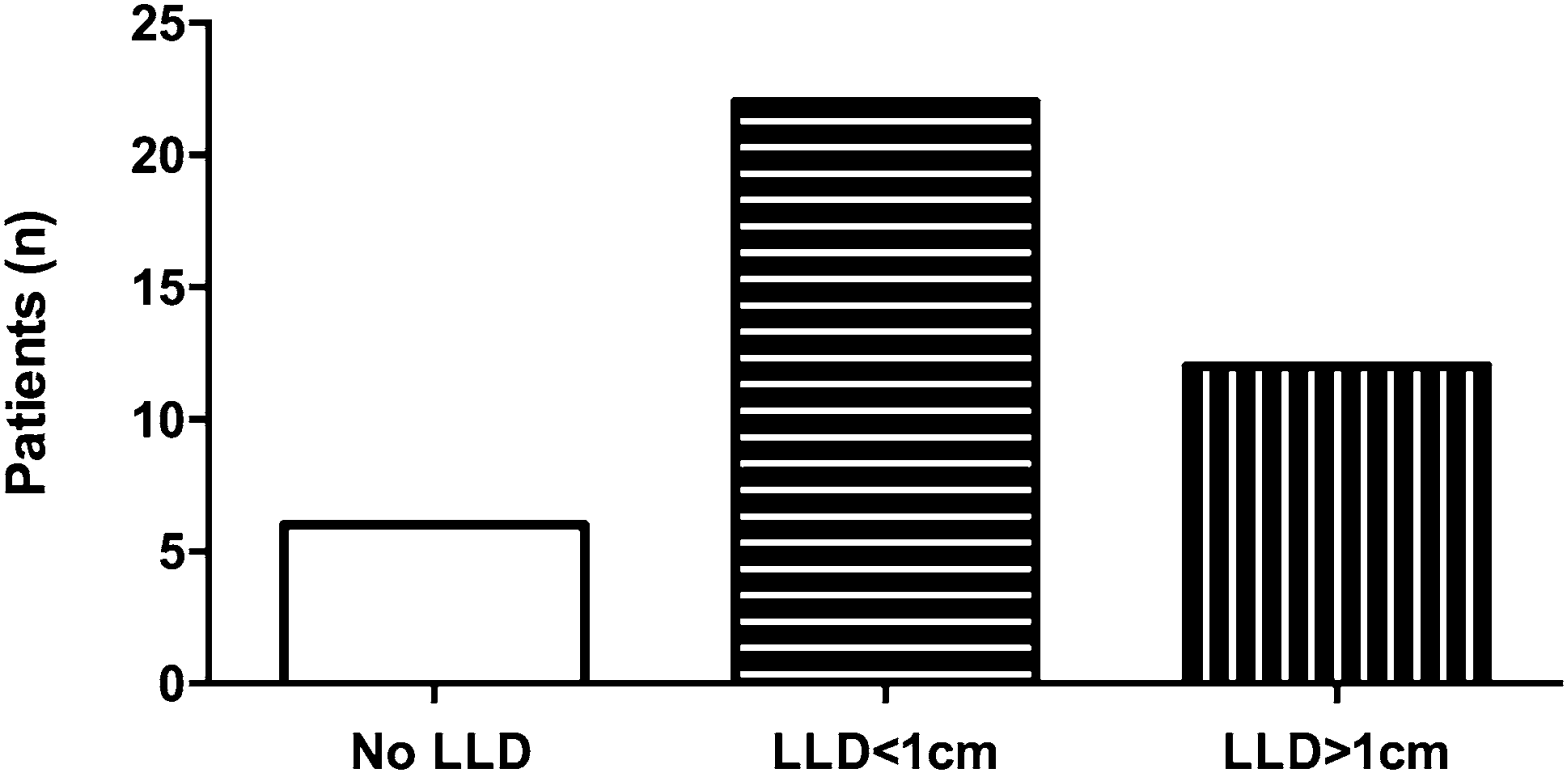
Overview of number of patients without leg length discrepancy (LLD) and with LLD of less than 10 mm and LLD of more than 10 mm at 5-year follow-up

**Fig. 4:**
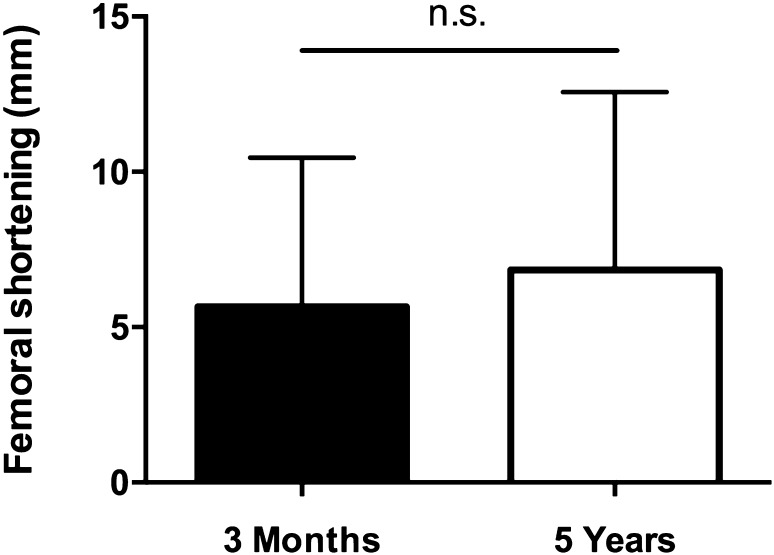
Comparison of femoral shortening in mm at both 3 months and 5 years followup, *n.s.* not significant

In obtained whole-leg standing X-rays, LLD was noticed in 34 patients (85%) with a median LLD of 7 mm. Twenty-two patients (65%) experienced LLD of less than 10 mm, and 12 patients (35%) of more than 10 mm (Fig. 3). No differences in extent of LLD between both genders and surgical techniques were observed. Again, fractures with Pauwels grade III led to significantly increased LLD (5 vs. 5 vs. 10 mm, *p* < 0.05). Type B2 fractures according to the AO/OTA classification caused significantly larger dimension of LLD compared to B1 and B3 fractures (B1: 5 mm, B2: 10 mm, B3: 5 mm, *p* < 0.05). No differences regarding Garden classification were found. Extent of LLD correlated with patient weight (*r* = 0.535, *p* < 0.05). We did not find any differences in surgery time, hospitalisation time, age, gender, trauma mechanism, and type of surgery. As expected, shortening of the femoral neck (*r* = 0.662, *p* < 0.001) as well as femoral shortening (*r* = 0.443, *p* > 0.01) at 3 months postoperatively correlated with LLD at invited follow-up. We did not observe significant increase of femoral shortening between 3 months and 5 years postoperative (Fig. 4).

#### Functional outcome at 5 years

Overall, the functional outcome was excellent with a median HHS of 96 (IQR: 90–100). 30 patients (75%) scored excellent results in the HHS, in 4 patients (10%) good results were found, in 2 patients (5%) fair results and in 4 patients (10%) poor results were documented. We did not see significant differences in functional outcome between gender and treatment.

We did not observe differences in outcome parameters associated with shortening of the femoral neck, femoral shortening or LLD. We found only a statistical trend towards lower HHS in patients with femoral shortening of 10 mm or more (93 vs. 99, *p* = 0.081). Other tested variables including, time of surgery, surgical technique, trauma mechanism, age, gender, fracture type, and hospitalisation time did not show any correlation with shortening of the femoral neck, femoral shortening, nor with occurrence or extent of LLD.

## Discussion

Shortening of the femoral neck following internal fixation of femoral neck fractures is a known phenomenon. However, the available literature describes incidence and extent of shortening in these patients of only up to 2 years [2, 10, 11]. Furthermore, these studies mainly did not focus on young patients, who due to higher functional demands, more likely suffer from shortening and LLD compared to elderly patients. Previous studies have shown that also younger patients are prone to experience shortening after fractures of the proximal femur [11–13]. Advocators of prosthetic replacement as treatment of femoral neck fractures often use shortening as argument against internal fixation of these fractures. We showed that following internal fixation of femoral neck fractures shortening is present in most cases, with a prevalence of 92.5% at 3 months postoperatively. In 32.5% of patients, femoral neck shortening of more than 10 mm was seen. Confirming our data, Zlowodzki et al. reported a prevalence of 30% of femoral neck shortening of more than 10 mm [10]. Also, median femoral neck shortening of 7 mm and median femoral shortening of 5 mm 3 months postoperatively were comparable with the available literature [2, 10]. Our data indicate that the fracture angle used in Pauwels classification might be of relevance for the extent of femoral neck shortening. We found that higher fracture angles were associated with larger extent of femoral neck shortening. Furthermore, weight was correlated to the extent of shortening. Both findings confirm results provided by Zielinski and colleagues. They showed that aside from Pauwels classification, also BMI and age were associated with shortening [2]. In our study a correlation with age was not observed. We, therefore, assume that in non-geriatric patients with lower rates of osteoporosis the correlation of age with femoral neck shortening might be negligible.

Since the available literature focused on shortening up to 2 years follow-up, we were interested whether incidence and extent of shortening increased in the mid-term follow-up and if there was a correlation with early shortening. The incidence of femoral neck shortening and femoral shortening was comparable at postoperative and invited follow-up. This suggests that shortening after femoral neck fractures in non-geriatric patients occurs mainly during the initial 3 months. As expected, the amount of initial shortening correlated with LLD at invited follow-up.

There is still the ongoing discussion, whether primary arthroplasty or internal fixation is the better treatment option in younger patients [4–6, 9, 13–15]. While internal fixation tries to preserve the joint, there is a significant risk of implant failure, revision surgery, and avascular femoral head necrosis following femoral neck fractures and internal fixation. Up to 27% salvage arthroplasty were reported in a previous study [9]. We observed a comparatively low rate of secondary hip replacement of 16%, which might be explained in part by the relatively high number of patients lost to follow-up. However, Slobogean et al. reported a re-operation rate of 14% due to avascular head necrosis, which is comparable to our findings [16]. Another study found an even lower rate with around 7% of avascular head necrosis [17]. Here presented data support internal fixation, which should be the primary treatment in young patients with femoral neck fractures. Even in cases of necessary secondary arthroplasty, potential gain in time can decrease chance of revision surgery after prosthetic replacement.

Importantly and further supporting primary internal fixation, we did not observe statistical significant differences in functional outcome in dependence of femoral shortening at 5-year follow-up. Only a trend towards lower HHS in patients experiencing more than 10 mm of femoral shortening was observed with still excellent scores in this sub-group. Other studies have shown that shortening was linked to impaired functional outcome [2, 10]. In these studies, however, the group of patients with larger extent of shortening were older compared to patients with less shortening. This potential bias might also have caused lower number in the HHS. Also, overall our patients were younger with potentially higher capacity to compensate for shortening.

Sub-group analysis revealed no relevant differences in terms of shortening and functional outcome. However, comparison between these two fixation methods has to be dealt with caution due to small patient numbers. Siavashi and colleagues found a lower rate of fixation failure of DHS compared to cancellous screws for the treatment of femoral neck fractures [18]. Another study failed to show significant differences between both treatment options [17]. Recently, the FAITH trial reported comparable outcomes for fracture fixation with the DHS and cannulated screws with small advantages in sub-group analyses favouring treatment with DHS [19].

Our postoperative mobilisation scheme consists of 3 months non-weight bearing if tolerated following internal fixation of femoral neck fractures. The rationale behind the non-weight-bearing mobilisation was to reduce pressure at the fracture site potentially reducing shortening and limiting the risk of AVN. However, we found a high incidence rate of femoral shortening and LLD in our patients. Furthermore, Wang et al. reported no correlation between postoperative mobilisation scheme and the risk for AVN [20]. Taken together, following these findings our postoperative non-weight-bearing regime has to be questioned.

Aside from impaired hip function, shortening with concomitant untreated LLD is a known predisposing factor for osteoarthritis of the knee, hip, and spine causing pain and disability [21]. In the overall population, LLD rates between 3 and 15% have been described [21]. In our study, we found a high overall rate of LLD of 85%, 5.5 years after surgery. Our data suggest that especially young patients benefit from long-term clinical follow-up.

## Conclusion

Taken together, our findings indicate that internal fixation in non-geriatric patients with femoral neck fractures is associated with excellent results despite high incidence of shortening at mid-term follow-up.

## Limitations

The major limitation of the present study is its retrospective design. Also, a comparatively low number of patients met our inclusion criteria (48 out of 163 patients, 29.5%). Main reason for exclusion was incomplete radiological follow-up available and concomitant severe injuries. This might have influenced our study collective. On the other hand, 40 patients out of 48 contacted patients (83.3%) returned for invited follow-up examination. We are also not able to determine whether congenital LLD played a role and influenced our findings.
